# Sex differences in time course and diagnostic accuracy of GFAP and UCH-L1 in trauma patients with mild traumatic brain injury

**DOI:** 10.1038/s41598-023-38804-4

**Published:** 2023-07-22

**Authors:** Linda Papa, Gretchen M. Brophy, Wilmer Alvarez, Robert Hirschl, Marshall Cress, Kurt Weber, Philip Giordano

**Affiliations:** 1 Department of Emergency Medicine, Orlando Health Orlando Regional Medical Center, 1335 Sligh Boul. 5th Floor, Orlando, FL 32806 USA; 2 Department of Pharmacotherapy and Outcomes Science and Neurosurgery, Virginia Commonwealth University, Richmond, VA USA; 3 Orlando Health Foundation, Orlando Health Orlando Health Regional Medical Center, Orlando, FL USA; 4 Department of Neurosurgery, Orlando Health Orlando Regional Medical Center, Orlando, FL USA

**Keywords:** Biomarkers, Neurology

## Abstract

Glial Fibrillary Acidic Protein (GFAP) and Ubiquitin C-terminal hydrolase (UCH-L1) have been FDA-approved for clinical use in mild and moderate traumatic brain injury (TBI). Understanding sex differences in their diagnostic accuracy over time will help inform clinical practice. We sought to evaluate the sex differences in the temporal profile of GFAP and UCH-L1 in a large cohort of trauma patients presenting to the emergency department. To compare the biomarkers’ diagnostic accuracy in male versus female patients for detecting mild TBI (MTBI), and traumatic intracranial lesions on head CT. This prospective cohort study enrolled female and male adult trauma patients presenting to a Level 1 Trauma Center. All patients underwent rigorous screening to determine whether or not they had experienced a MTBI. Of 3025 trauma patients assessed, 1030 met eligibility criteria and 446 declined. Initial blood samples were obtained in 584 patients enrolled within 4 h of injury. Repeated blood sampling was conducted at 4, 8, 12, 16, 24, 36, 48, 60, 72, 84, 96, 108, 120, 132, 144, 156, 168, and 180-h post-injury. The main outcomes included the diagnostic accuracy in detection of MTBI and traumatic intracranial lesions on head CT scan. A total of 1831 samples were drawn in 584 patients over 7 days, 362 (62%) were male and 222 (38%) were female. The pattern of elevation was similar in both sexes. Although the pattern of elevation was similar between male and female for both biomarkers, male patients had significantly higher concentrations of UCH-L1 compared to female patients at several timepoints post-injury, particularly within 24 h of injury. There were no significant differences in diagnostic accuracy for detecting MTBI or for detecting CT lesions between male and female patients at any timepoint for both GFAP and UCH-L1. Although patterns of GFAP and UCH-L1 release in trauma patients over a week post-injury was similar between the sexes, there were significantly higher concentrations of UCH-L1 in males at several timepoints post-injury. Despite this, the overall diagnostic accuracies of both GFAP and UCH-L1 over time for detecting MTBI and CT lesions were not significantly different between male and female trauma patients.

## Introduction

A blood test for traumatic brain injury (TBI) has been FDA-approved in the United States for adult patients with mild to moderate TBI to help determine the need for CT scan of the head within 12 h of injury^1^. The test is comprised of two biomarkers: (1) Glial Fibrillary Acidic Protein (GFAP), an astroglial marker of injury and is found in the astroglial skeleton of both white and gray brain matter and, (2) Ubiquitin Carboxy-terminal Hydrolase-L1 (UCH-L1), a neuronal brain injury marker found in high abundance in neurons. Both GFAP and UCH-L1 have been evaluated in several studies to detect acute traumatic intracranial lesions on computed tomography (CT) scan following a mild to moderate TBI in adults^2–11^. A number of articles have also described how GFAP and UCH-L1 are able to detect mild TBI in trauma patients^2,4,5^.

Despite the volume of TBI biomarker research, clinical TBI biomarker research has failed to adequately examine sex differences between male and female TBI patients with respect to their ability to detect significant brain injury^12^. Studies have started to assess biomarker sex differences in chronic TBI outcomes^13^. It remains unresolved whether the diagnostic accuracy of these biomarkers acutely and sub-acutely is similar in men and women and if they should be interpreted differently. This is particularly important in mild TBI patients where concentrations are often close to the level of detection. With such low biomarker values, it is important to develop reference values for proper clinical interpretation.

This study addressed these shortfalls by evaluating sex differences in circulating serum glial and neuronal serum biomarkers GFAP and UCH-L1 over 7 days post-injury in a large cohort of trauma patients presenting to the emergency department. The objectives included comparing time course patterns of these markers in male and female patients following trauma, as well as assessing their diagnostic accuracy for detecting MTBI and traumatic intracranial lesions on head CT at several timepoints over 7 days. The focus of our study was on MTBI (99% of patients had a GCS score 13–15 and 92% had a GCS score of 15). Sex differences were defined by biological attributes that distinguished males and females based on reproductive organs and chromosomes complement rather than gender which reflects non-biological traits, behaviors, and expectations ascribed to men and women^14^.

## Methods

### Study population

This is a secondary analysis of a prospective cohort study which enrolled a convenience sample of adult trauma patients presenting to the Emergency Department (ED) of a Level I Trauma Center in Orlando, Florida within 4 h of injury^5^. Eligibility for mild to moderate TBI was determined by the treating physician based on the history of blunt head trauma followed by either loss of consciousness, amnesia, or disorientation and presenting to the emergency department within 4 h of injury with a GCS of 9 to 15.

The reason some patients with 9–12 were considered for enrollment was because mild and moderate TBI are often difficult to assess and distinguish clinically during the first hours after injury if patients are intoxicated, medicated, in emotional distress, or in severe pain. Our goal was to enroll a mild trauma population with both mild TBI and mild trauma control patients (99% of our patients had a GCS score of 13–15 with a median ISS score 4).

Prior to enrollment the research team carefully verified eligibility. Head CT Scans were not required and were performed at the discretion of the treating physician. Exclusion criteria comprised of patients who: (1) were less than 18 years old; (2) had no history of trauma as their primary event (e.g., syncope or seizure); (3) had known dementia, chronic psychosis or active CNS pathology; (4) were pregnant; or (5) were incarcerated or (6) had a systolic blood pressure less than 100 mmHg.

The non-TBI general trauma group (trauma controls) included patients with GCS 15 presenting to the emergency department with a traumatic mechanism of injury but without TBI. They experienced similar mechanisms of injury as the MTBI group, but all had a normal mental status since injury (as verified by the research team) and had no evidence of acute brain injury or hemodynamic instability. These patients were carefully screened to ensure they had no loss of consciousness, no amnesia and no alteration in sensorium at any time after injury. The purpose of enrolling both TBI and general trauma patients was to simulate the real world setting in which TBI biomarkers would be used.

This study was approved by the Institutional Review Board (IRB) and written informed consent was obtained from each patient and/or their legal authorized representative prior to enrollment. All methods were performed in accordance with US Federal regulations for protection of human subjects.

### Study procedures

All initial patient assessments were made by board certified emergency medicine physicians trained by a formal one-hour session on evaluating patient eligibility for the study. Following the initial screening, a meticulous secondary assessment was conducted by the research team prior to enrollment to ensure each patient strictly met inclusion and exclusion criteria. All prehospital and emergency department records were reviewed, patients, families and witnesses were carefully questioned (if available), and the final determination was made by the emergency physician together with the research team. Patient classification was performed prospectively, not retrospectively.

Blood samples were obtained from each MTBI and trauma patient within 4 h of the reported time of injury. Repeated blood sampling was conducted for as long as the patient remained in hospital at 4, 8, 12, 16, 24, 36, 48, 60, 72, 84, 96, 108, 120, 132, 144, 156, 168 and 180 h after injury. Once patients were discharged, no further blood samples were taken. After assessment and treatment in the emergency department, patients were either discharged home or admitted to hospital based on severity of their injuries and patient management was not altered by the study.

For each blood draw a single vial of approximately 5 mL of blood was collected and placed in serum separator tubes and allowed to clot at room temperature. The blood was centrifuged within 30 min and the serum was placed in bar-coded aliquot containers and stored in a freezer at − 70 °C until it was transported to a central laboratory. There, the samples were analyzed in batches using sandwich enzyme-linked immunosorbent assays (ELISA) to GFAP and UCH-L1. Lab personnel running the samples were blinded to the clinical data.

Trauma patients underwent standard CT scan of the head based on the clinical judgment of the treating physician. Most patients with blunt head trauma with subsequent symptoms had a head CT scan performed as part of usual care but it was not dictated as part of the study. Physicians often ordered CT scans of the head on the general trauma controls based on mechanism or clinical circumstances. CT examinations were interpreted by board-certified radiologists who recorded location, extent and type of brain injury. Radiologists were blinded to the study protocol but had the usual clinical information.

### Outcome measures

Performance of GFAP and UCH-L1 was evaluated over a 7-day period in detecting brain injury and compared in male versus female patients. Although sex and gender are often used interchangeably, we used the term “sex” as it includes the biological attributes based on reproductive organs and chromosomes complement (gender reflects non-biological traits and behaviors ascribed to men and women)^14^. The main outcome measures included the performance of the biomarkers over time for: (1) detecting the presence of MTBI and in distinguishing trauma patients with MTBI from those without MTBI, and (2) detecting traumatic intracranial lesions on CT scan.

Intracranial lesions on CT included any acute traumatic intracranial lesions visualized on CT scan as defined by any traumatic intracranial lesion including intracranial hemorrhage (epidural, subdural, subarachnoid hemorrhage) or contusion, cerebral edema, diffuse axonal injury, midline shift of intracranial contents or signs of brain herniation, or pneumocephalus. Number, type and severity of lesions were reported, and the Rotterdam CT classification score was calculated for each patient with traumatic intracranial lesions. The score includes four independently scored elements: (1) degree of basal cistern compression, (2) degree of midline shift, (3) epidural hematomas, (4) intraventricular and/or subarachnoid blood. A completely normal appearing scan has a Rotterdam score of 1 and the worst possible score is 6.{Maas, 2005 #5609}{Talari, 2016 #5608}.

### Statistical analysis

Descriptive statistics with means and proportions were used to describe the data. For statistical analysis, biomarker concentrations were treated as continuous data, measured in ng/ml and expressed as medians with interquartile range. Data were assessed for equality of variance and distribution. Logarithmic transformations were conducted on non-normally distributed data. Group comparisons were performed using independent sample t-test with variance consideration and the chi-squared test. Receiver Operating Characteristics (ROC) curves were created to explore the ability of the biomarkers to identify the presence of a TBI versus trauma controls and to detect intracranial lesions on CT scan. Estimates of the area under these curves (AUROC) were obtained (AUROC = 0.5 indicates no discrimination and an AUROC = 1.0 indicates a perfect diagnostic test). Classification performance was assessed by sensitivity, specificity, positive and negative predictive values with 95% confidence intervals. Comparisons of ROC curves between sexes were performed using the technique by Hanley and McNeil^15,16^.

Logistic regression analysis was conducted on the entire patient cohort to adjust for potential injury severity differences between female and male patients such as age, GCS score, injury severity score relative to biomarker concentrations. Generalized estimating equation (GEE) models were used for longitudinal analysis of correlated data to assess the association of age, sex, injury severity and GCS scores with UCH-L1 concentrations over time post-injury. The model included UCH-L1 concentrations as the dependent variable. Independent variables included age, sex, injury severity score, and GCS score with an assessment of interaction between sex and time. An autoregressive working correlation matrix was used. Repeated variables included subjects and timepoints. Data were presented as odds ratios and 95%CIs. All analyses were performed using the statistical software package SPSS 28.0 (IBM Corporation®, Somers NY).

### Biomarker analysis

Serum GFAP and UCH-L1 concentrations were measured in duplicate for each sample using a validated ELISA platform (Banyan Biomarkers Inc., Alachua Florida USA). For the GFAP assay, the lower limit of quantification (LLOQ) is 0.030 ng/ml and upper limit of quantification (ULOQ) is 50 ng/ml. The limit of detection (LoD) is 0.008 ng/mL. For the UCH-L1 assay, the lower limit of quantification (LLOQ) is 0.100 ng/ml and upper limit of quantification (ULOQ) is 9 ng/ml. The limit of detection (LoD) is 0.045 ng/mL. Any samples yielding a signal over the quantification or calibrator range were diluted and re-assayed.

### Ethical approval

This study was approved by the Orlando Regional Medical Center Institutional Review Board in accordance with Federal Regulations for Research in the United States of America.

### Transparency

The lead author (LP) affirms that this manuscript is an honest, accurate, and transparent account of the study being reported; that no important aspects of the study have been omitted.

## Results

**Figure 1:**
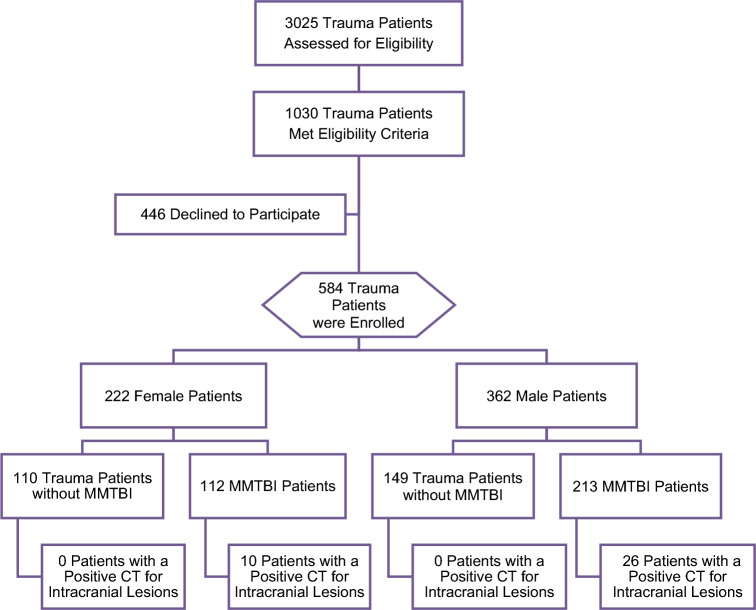
Flow diagram of screened and enrolled patients.

Of 3025 trauma patients screened, 1030 patients met eligibility criteria, and 584 trauma patients were enrolled: 362 (62%) were male and 222 (38%) were female. The flow diagram in Fig. 1 describes the distribution of enrolled patients. Demographic characteristics between enrolled and non-enrolled patients were similar. Enrolled patients had a mean age in years of 40 (SD16) [range 18–83] and non-enrolled patients were 41 (SD17) [range 18–88] (*p* = 0.39). The proportion of males and females in the enrolled and non-enrolled patients was similar (*p* = 0.48) and race was not significantly different (*p* = 0.07). Of those enrolled, 325 (56%) had trauma with MTBI and 259 (44%) had trauma without TBI (trauma controls). Among patients with TBI, 98% were mild and had a GCS score of 13–15. CT scans of the head were performed in 315 (97%) patients with MTBI and in 97 (37%) of trauma patients without MTBI. All trauma patients without MTBI had a GCS score of 15. Intracranial lesions were found in 36 patients who had CT scans (8.7%). All CT lesions were found in MTBI patients, and none were found trauma patients without MTBI. Of the seven patients who presented with an initial GCS of 9–12, four had no lesions on CT scan. The distribution of clinical characteristics of all enrolled patients is presented in Table 1. There were no significant differences in traumatic intracranial lesion number, type or severity between male and female patients. The mean Rotterdam Scores was 1.7 in both sexes (1 = normal and 6 = worst) (Table 2), signifying injuries with better prognosis.

**Table 1:** Characteristics of enrolled patients.

Characteristics	Male patients	Female patients	Total
	N = 362	N = 222	N = 584
Mean age (yrs ± SD)	40 (± 15)	40 (± 17)	40 (± 16)
Age Range	(18–83)	(18–79)	(18–83)
Race (%)			
Asian	5 (1)	3 (1)	8 (1)
Black	73 (20)	60 (27)	133 (23)
Hispanic	79 (22)	39 (18)	118 (20)
Native American	2 (1)	2 (1)	4 (1)
Middle Eastern	1 (< 1)	0 (0)	1 (< 1)
White	196 (54)	114 (51)	310 (53)
Other	6 (2)	4 (2)	10 (2)
GCS Score in ED (%)			
GCS 9–12	5 (2)	2 (1)	7 (1)
GCS 13	1 (< 1)	2 (1)	3 (1)
GCS 14	25 (7)	14 (6)	39 (7)
GCS 15	331 (91)	204 (92)	535 (92)
Mechanism of Injury (%)			
Motor Vehicle Crash	142 (39)	158 (71)	300 (51)
Fall	74 (20)	25 (11)	99 (17)
Motorcycle	52 (14)	5 (2)	57 (10)
Pedestrian Struck	12 (3)	7 (3)	19 (3)
Bicycle Struck by Vehicle	20 (6)	3 (1)	23 (4)
Fall off Bicycle	6 (2)	2 (1)	8 (1)
Assault	9 (3)	10 (5)	19 (3)
Sports Injury	6 (2)	4 (2)	10 (2)
Other Motorized Vehicle	4 (1)	1 (1)	5 (1)
Other	37 (10)	7 (3)	44 (8)
Loss of Consciousness (%)	182 (50)	86 (39)	268 (46)
Amnesia (%)	87 (24)	54 (24)	141 (24)
ISS (median-IQR) (N = 558)	5 (2–8)	4 (2–6)	4 (2–8)
Admitted to Hospital (%)	136 (38)	53 (24)	189 (32)
CT Head performed (%)	270 (75)	144 (65)	414 (71)
Intracranial Lesions on head CT (%)	26 (10)	10 (7)	36 (9)
Neurosurgical Intervention (%)	4 (2)	3 (2)	7 (2)

Due to rounding, percentages may not add up to 100.

**Table 2:** Description of Traumatic Intracranial CT Head Lesions.

Characteristics	Male patients	Female patients	Total	*p*-value
	N = 26	N = 10	N = 36	
Intracranial lesions on head CT*				
Subarachnoid hemorrhage	12 (46%)	6 (60%	18 (50%)	0.711
Subdural hematoma	10 (39%)	4 (40%)	14 (39%)	0.999
Epidural hematoma	2 (8%)	0 (0)	2 (6%)	0.999
Contusion/parenchymal hemorrhage	8 (31%)	2 (20%)	10 (28%)	0.689
Traumatic axonal injury/petechia	3 (12%)	3 (30%)	6 (17%)	0.317
Pneumocephalus	3 (12%)	2 (20%)	5 (14%)	0.603
Midline shift	1 (4%)	1 (10%)	2 (6%)	0.484
Basal skull fracture	3 (12%)	3 (30%)	6 (17%)	0.317
Skull fracture (depressed/comminuted)	7 (27%)	3 (30%	10 (28%)	0.999
Number of different lesions				
1	10 (39%)	3 (30%)	13 (36%)	
2	10 (39%)	3 (30%)	13 (36%)	
3	5 (19%)	2 (20%)	7 (19%)	0.496
4	1 (4%)	1 (10%)	2 (6%)	
5	0 (0)	1 (10%)	1 (3%)	
Rotterdam CT score				
Rotterdam score	1.7 (1.4–1.9)	1.7 (1.2–2.2)	1.7 (1.5–1.9)	0.974
Risk of 6-month mortality	5% (3.1–6.9)	5.1% (1.4–8.8)	5 (3.4–6.6)	0.956

*Some patients have more than one lesion type.

There were a total of 1831 samples drawn in 584 patients (1215 samples in males and 622 in females). The average time from injury to serum sample collection was 3 h (SD 0.8): 3 h (SD 0.85) for MTBI patients and 3.1 h (SD 0.75) trauma control patients. All patients (584) had samples drawn between injury and 4 h, 429 patients had samples taken at 4-h post-injury, 136 at 8-h, 107 at 12-h, 96 at 16-h; 88 at 20-h, 81 at 24-h, 57 at 36-h, 50 at 48-h, 41 at 60-h, 38 at 72-h, 28 at 84-h, 25 at 96-h, 13 at 108-h, 13 at 120-h, 10 at 132-h, 12 at 144-h, 11 at 156-h, and 8 at 168-h, and 4 at 180-h post-injury.

### Temporal profile all trauma patients

**Figure 2:**
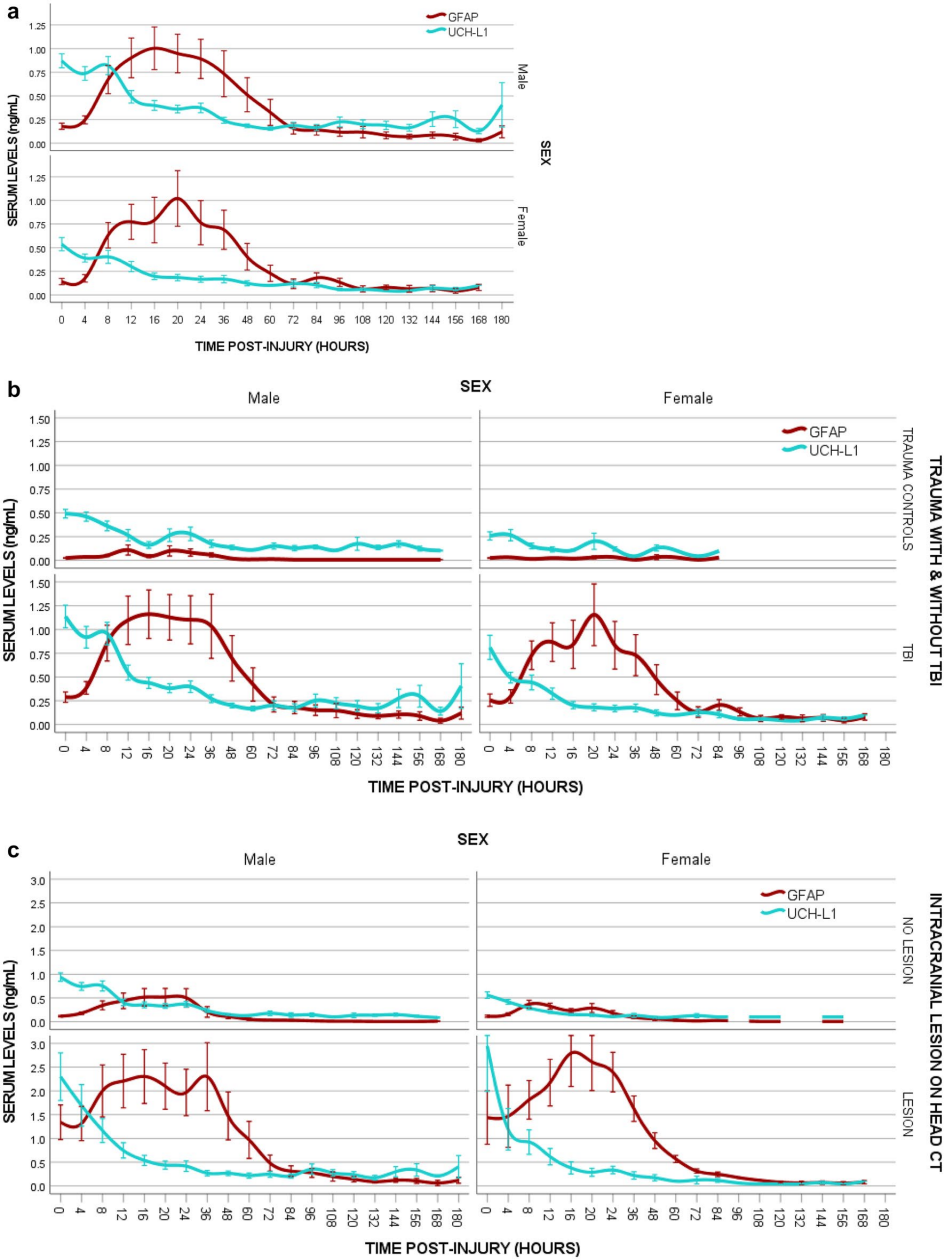
(**a**) Comparison of the Temporal Profiles of GFAP and UCH-L1 in Males versus Females all Trauma patients. Means with error bars representing standard error of the mean (SEM). (**b**) Comparison of the Temporal Profile of GFAP and UCH-L1 in Males versus Females in trauma patients with and without MTBI. Temporal Profile of GFAP and UCH-L1 in trauma patients who had clinical evidence of a mild TBI compared to those who experienced a trauma but had no evidence of a mild TBI. Means with error bars representing standard error of the mean (SEM). (**c**) Comparison of the Temporal Profile of GFAP and UCH-L1 in Males versus Females in trauma patients with and without traumatic intracranial lesions on Head CT. Temporal Profile of GFAP and UCH-L1 in trauma patients who had a CT performed. Those with traumatic intracranial lesions on CT are compared to those without intracranial lesions on CT. Means with error bars representing standard error of the mean (SEM).

A comparison of the time course of GFAP and UCH-L1 in male and female patients with trauma is shown in Fig. 2a. In both male and female patients, the concentration of GFAP was detectible within 1-h of injury and reached a peak at 20-h post-injury. Concentrations steadily decreased over 72 h. GFAP levels were still detectable at 168-h post-injury and remained at lower levels between 72- and 180-h post-injury. There were no significant differences in concentration of GFAP between males and female patients at any timepoint after injury (eTable 1a). With UCH-L1, the pattern of elevation was similar between male and female patients with a peak at 8 h and steady decline over 48 h. However, male patients had significantly higher levels of UCH-L1 than female patients at several timepoints post-injury, particularly within 72 h of injury with univariate analysis (eTable 1b).

When factors such as GFAP and UCH-L1 concentrations, age, injury severity score, and GCS Score at ED arrival were modeled using logistic regression analysis relative to sex, elevated UCH-L1 concentrations were significantly associated with the male sex in all patients, as well as in the MTBI, and trauma control cohorts (eTable 2). When GEE models were used to assess the association of age, sex, injury severity score, and GCS Score on UCH-L1 concentrations longitudinally over 7 days, sex was a significant factor in the model in the entire cohort, as well as in the MTBI, and trauma control cohorts (eTable 3). Furthermore, there were significant interactions between sex and time relative to UCH-L1 concentrations over 24 h in all patients (eTable 4).

### Temporal profile for detecting MTBI

A comparison of the time course of GFAP and UCH-L1 in male and female patients with and without MTBI (MTBI versus trauma controls) is shown in Fig. 2b. There were similar patterns of GFAP and UCH-L1 biomarker expression in male and female patients with and without MTBI. In patients *with MTBI* there were no significant differences in concentrations of GFAP between male and female patients at any timepoint after injury. However, there were significant differences in UCH-L1 concentrations between male and female patients on univariate analysis in patients *with MTBI* at several timepoints, including at enrollment (*p* = 0.033), at 4-h post-injury (*p* = 0.039), at 8-h (*p* = 0.004), at 16-h (*p* = 0.010), at 20-h (*p* = 0.010), at 24-h (*p* = 0.017), at 36-h (*p* = 0.022), at 48-h (*p* = 0.014), at 96-h (*p* = 0.008), and at 108-h post-injury (*p* = 0.008).

Potential injury severity differences between female and male patients in the MTBI group were assessed relative to biomarker concentrations. After adjusting for age, injury severity score, and GCS Score at ED arrival, GFAP concentrations were not significantly different between male and female patients but UCH-L1 concentrations showed significant differences between the sexes with higher levels in male patients (eTable 2). When GEE models were used to assess the association of age, sex, injury severity score, and GCS Score on UCH-L1 concentrations longitudinally over 7 days, sex was a significant factor in the model for the MTBI patients (eTable 3) and there were significant interactions between sex and time relative to UCH-L1 concentrations from enrollment, 4-, 8-, 12-, 16-, 20-, and 24-h post-injury (eTable 5).

In trauma patients *without MTBI* (trauma controls) there were no significant differences in concentrations of GFAP between male and female patients at any timepoint after injury but there were significant differences in UCH-L1 concentrations at enrollment (*p* < 0.001), 4-h post-injury (*p* < 0.001), and at 8-h post-injury (*p* = 0.012) on univariate analysis. After adjusting for age and injury severity score at ED arrival, GFAP concentrations were not significantly different between male and female patients but UCH-L1 concentrations showed significant differences between the sexes with higher levels in male patients (eTable 2). When GEE models were used to assess the association of age, sex, injury severity score, and GCS Score on UCH-L1 concentrations longitudinally over 7 days, sex was a significant factor in the model for the trauma control patient with no MTBI (eTable 3) and there were significant interactions between sex and time relative to UCH-L1 concentrations from enrollment, 4-, 8-, 12-, 16-, 20-, and 24-h post-injury (eTable 6).

### Temporal profile for detecting intracranial lesions on CT

Figure 2c compares the time course of GFAP and UCH-L1 in male and female patients with and without traumatic intracranial lesions on CT scan. There were similar patterns of GFAP and UCH-L1 biomarker expression among male and female patients with and without intracranial lesions. There were no statistically significant differences in concentrations of GFAP between male and female patients at any timepoint after injury in both those with and without intracranial lesions on CT. In contrast, among patient with *no lesions on CT*, concentrations of UCH-L1 were significantly higher in male compared to female patients at enrollment (*p* < 0.001), at 4-h post-injury (*p* < 0.001), at 8-h (*p* < 0.001), at 16-h (*p* = 0.037), at 20-h (*p* = 0.046), at 24-h (*p* = 0.009), and at 48-h (*p* = 0.039) post-injury. However, among patients *with lesions on CT scan*, UCH-L1 concentrations did not differ significantly between male and female patients.

### Diagnostic accuracy for detecting MTBI

Diagnostic accuracy of GFAP and UCH-L1 for detecting MTBI among all trauma patients was compared in male versus female patients using AUROC. There was a trend for AUROCs for GFAP to be higher in female compared to male patients between 8 to 36 h post-injury but was only statistically significant at 8-h post-injury 0.90 versus 0.73 (*p* = 0.027) (Table 3). There was also a trend in AUROCs for UCH-L1 to be higher in female than male patients within 12-h of injury, but the differences were not statistically significant (Table 4).

**Table 3:** Area Under the ROC Curve for GFAP in distinguishing between trauma patients with and without MTBI (ability to detect MTBI among a cohort of trauma patients).

Time post-injury N = + MTBI/ − MTBI	GFAP (Male) N = 362	GFAP (Female) N = 222	GFAP (All) N = 584	*P*-Value
Enrollment	0.73 (0.68–0.78)	0.75 (0.69–0.82)	0.73 (0.69–0.77)	0.631
Male n = 213/149				
Female n = 112/110				
4 h	0.71 (0.65–0.77)	0.75 (0.67–0.82)	0.73 (0.68–0.77)	0.415
Male n = 162/106				
Female n = 88/73				
8 h	0.73 (0.63–0.83)	0.90 (0.81–1.00)	0.78 (0.70–0.86)	0.027
Male n = 71/21				
Female n = 38/6				
12 h	0.73 (0.61–0.85)	0.87 (0.75–0.99)	0.77 (0.68–0.87)	0.158
Male n = 57/14				
Female n = 32/4				
16 h	0.81 (0.70–0.91)	0.89 (0.76–1.00)	0.83 (0.74–0.91)	0.425
Male n = 55/9				
Female n = 30/2				
20 h	0.78 (0.66–0.91)	0.82 (0.64–0.99)	0.79 (0.69–0.90)	0.750
Male n = 52/11				
Female n = 22/3				
24 h	0.79 (0.68–0.91)	0.81 (0.63–0.99)	0.80 (0.70–0.90)	0.891
Male n = 46/12				
Female n = 21/2				
36 h	0.80 (0.66–0.94)	0.91 (0.75–1.00)	0.81 (0.70–0.93)	0.388
Male n = 27/12				
Female n = 17/1				
48 h	0.90 (0.80–1.00)	0.77 (0.49–1.00)	0.86 (0.76–0.96)	0.452
Male n = 27/10				
Female n = 11/2				
60 h	0.94 (0.85–1.00)	0.60 (0.28–0.92)	0.88 (0.78–0.99)	0.250
Male n = 23/7				
Female n = 10/1				
72 h	0.84 (0.69–0.99)	0.88 (0.61–1.00)	0.84 (0.71–0.97)	0.813
Male n = 21/8				
Female n = 8/1				
84 h	0.94 (0.83–1.00)	0.83 (0.54–1.00)	0.90 (0.79–1.00)	0.597
Male n = 16/5				
Female n = 6/1				
96 h	0.84 (0.68–1.00)	n/a	0.85 (0.70–1.00)	–
Male n = 16/5				
Female n = 4/0				
108 h	0.86 (0.60–1.00	n/a	0.82 (0.57–1.00)	–
Male n = 7/2				
Female n = 4/0				
120 h	0.88 (0.66–1.00)	n/a	0.90 (0.73–1.00)	–
Male n = 8/3				
Female n = 2/0				
132 h	0.92 (0.71–1.00)	n/a	0.94 (0.78–1.00)	–
Male n = 6/2				
Female n = 2/0				
144 h	0.88 (0.65–1.00)	n/a	0.90 (0.72–1.00)	–
Male n = 8/2				
Female n = 2/0				
156 h	0.83 (0.54–1.00)	n/a	0.83 (0.58–1.00)	–
Male n = 6/2				
Female n = 3/0				
168 h*	0.81 (0.53–1.00)		0.85 (0.62–1.00)	–
Male n = 8/2				
Female n = 2/0				

Shown is the performance of GFAP in males versus females.

*Includes results from 180 h.

**Table 4:** Area under the ROC Curve for UCH-L1 in distinguishing between trauma patients with and without TBI ((ability to detect MTBI among a cohort of trauma patients).

Time post-Injury N = + MTBI/ − MTBI	UCH-L1 (Male) N = 362	UCH-L1 (Female) N = 222	UCH-L1 (All) N = 584	*P*-Value
Enrollment	0.62 (0.56–0.67)	0.70 (0.64–0.77)	0.66 (0.62–0.70)	0.079
Male n = 213/149				
Female n = 112/110				
4 h	0.59 (0.52–0.66)	0.69 (0.61–0.77)	0.63 (0.58–0.69)	0.064
Male n = 162/106				
Female n = 88/73				
8 h	0.66 (0.55–0.78)	0.74 (0.57–0.91)	0.65 (0.55–0.75)	0.485
Male n = 71/21				
Female n = 38/6				
12 h	0.62 (0.48–0.76)	0.78 (0.62–0.95)	0.63 (0.51–0.75)	0.217
Male n = 57/14				
Female n = 32/4				
16 h	0.72 (0.57–0.87)	0.66 (0.45–0.87)	0.67 (0.53–0.81)	0.762
Male n = 55/9				
Female n = 30/2				
20 h	0.59 (0.42–0.76)	0.41 (0.13–0.68)	0.53 (0.38–0.68)	0.382
Male n = 52/11				
Female n = 22/3				
24 h	0.56 (0.39–0.73)	0.48 (0.20–0.76)	0.52 (0.37–0.67)	0.737
Male n = 46/12				
Female n = 21/2				
36 h	0.66 (0.47–0.85)	0.91 (0.75–1.00)	0.61 (0.44–0.78)	0.073
Male n = 27/12				
Female n = 17/1				
48 h	0.65 (0.46–0.85)	0.32 (0–0.66)	0.58 (0.40–0.75)	0.184
Male n = 27/10				
Female n = 11/2				
60 h	0.67 (0.48–0.85)	0.65 (0.25–1.00)	0.60 (0.43–0.77)	0.946
Male n = 23/7				
Female n = 10/1				
72 h	0.52 (0.30–0.74)	0.81 (0.48–1.00)	0.52 (0.31–0.72)	0.218
Male n = 21/8				
Female n = 8/1				
84 h	0.51 (0.25–0.77)	0.42 (0.01–0.82)	0.50 (0.28–0.72)	0.808
Male n = 16/5				
Female n = 6/1				
96 h	0.49 (0.23–0.75)	n/a	0.40 (0.17–0.63)	–
Male n = 16/5				
Female n = 4/0				
108 h	0.63 (0.29–0.96)	n/a	0.42 (0.14–0.70)	–
Male n = 7/2				
Female n = 4/0				
120 h	0.52 (0.16–0.88)	n/a	0.42 (0.10–0.73)	–
Male n = 8/3				
Female n = 2/0				
132 h	0.42 (0.03–0.81)	n/a	0.31 (0–0.63)	–
Male n = 6/2				
Female n = 2/0				
144 h	0.38 (0.04–0.71)	n/a	0.30 (0.02–0.58)	–
Male n = 8/2				
Female n = 2/0				
156 h	0.71 (0.35–1.00)	n/a	0.50 (0.17–0.83	–
Male n = 6/2				
Female n = 3/0				
168 h*	0.72 (0.40–1.00)	n/a	0.63 (0.30–0.96)	–
Male n = 8/2				
Female n = 2/0				

Shown is the performance of UCH-L1 in males versus females.

*Includes results from 180 h.

### Diagnostic accuracy for detecting intracranial lesions on CT

The ability of GFAP to detect traumatic intracranial lesions on CT scan followed a comparable trend, with AUROCs being higher in female patients compared to male patients (Table 5). The differences, however, were not statistically significant except at 24-h post-injury (*p* = 0.005). Analogously, AUROCs to detect traumatic intracranial lesions on CT scan for UCH-L1 tended to be higher in female patients compared to male patients but did not reach statistical significance (Table 6).

**Table 5:** Area Under the ROC Curve for GFAP in distinguishing between trauma patients with and without Intracranial Lesions on CT Scan of the head (ability to detect CT lesions).

Time post-injury N = CT + /CT-	GFAP (Male) N = 270	GFAP (Female) N = 144	GFAP (All) N = 414	*P*-Value
Enrollment	0.86 (0.77–0.94)	0.88 (0.77–1.00)	0.86 (0.79–0.93)	0.810
Male n = 26/244				
Female n = 10/134				
4 h	0.82 (0.72–0.92)	0.89 (0.73–1.00)	0.84 (0.75–0.92)	0.476
Male n = 24/187				
Female n = 7/107				
8 h	0.79 (0.66–0.92)	0.88 (0.73–1.00)	0.81 (0.70–0.91)	0.376
Male n = 19/69				
Female n = 9/31				
12 h	0.80 (0.66–0.94)	0.89 (0.73–1.00)	0.82 (0.71–0.93)	0.369
Male n = 19/50				
Female n = 9/25				
16 h	0.77 (0.63–0.92)	0.86 (0.61–1.00)	0.81 (0.68–9.31)	0.447
Male n = 18/44				
Female n = 7/23				
20 h	0.77 (0.61–0.92)	0.88 (0.67–1.00)	0.80 (0.67–0.92)	0.324
Male n = 18/42				
Female n = 8/15				
24 h	0.76 (0.59–0.92)	0.99 (0.99–1.00)	0.82 (0.70–0.95)	0.005
Male n = 16/40				
Female n = 6/16				
36 h	0.95 (0.89–1.00)	0.99 (0.99–1.00)	0.97 (0.92–1.00)	0.483
Male n = 10/27				
Female n = 7/11				
48 h	0.94 (0.87–1.00)	0.99 (0.99–1.00)	0.96 (0.90–1.00)	0.419
Male n = 11/25				
Female n = 5/8				
60 h	0.96 (0.89–1.00)	0.99 (0.99–1.00)	0.97 (0.93–1.00)	0.621
Male n = 9/19				
Female n = 4/7				
72 h	0.93 (0.82–1.00)	0.99 (0.99–1.00)	0.95 (0.88–1.00)	0.451
Male n = 8/19				
Female n = 3/5				
84 h	0.91 (0.78–1.00)	0.99 (0.99–1.00)	0.94 (0.86–1.00)	0.367
Male n = 8/12				
Female n = 5/1				
96 h	0.88 (0.71–1.00)	n/a	0.91 (0.78–1.00)	–
Male n = 8/13				
Female n = 3/0				
108 h	0.85 (0.58–1.00)	0.99 (0.99–1.00)	0.90 (0.71–1.00)	0.361
Male n = 5/4				
Female n = 2/1				
120 h	0.88 (0.67–1.00)	n/a	0.91 (0.75–1.00)	–
Male n = 6/5				
Female n = 2/0				
132 h	0.92 (0.71–1.00)	n/a	0.94 (0.78–1.00)	–
Male n = 6/2				
Female n = 2/0				
144 h	0.93 (0.76–1.00)	n/a	0.94 (0.81–1.00)	–
Male n = 7/3				
Female n = 2/0				
156 h	0.90 (0.66–1.00)	0.99 (0.99–1.00)	0.93 (0.76–1.00)	0.512
Male n = 5/3				
Female n = 2/1				
168 h*	0.79 (0.50–1.00)	n/a	0.84 (0.53–1.00)	–
Male n = 6/4				
Female n = 2/0				

Shown is the performance of GFAP in males versus females.

*Includes results from 180 h.

**Table 6:** Area Under the ROC Curve for UCH-L1 in distinguishing between trauma patients with and without Intracranial Lesions on CT Scan of the head (ability to detect CT lesions).

Time post-injury N = CT + /CT−	UCH-L1 (Male) N = 270	UCH-L1 (Female) N = 144	UCH-L1 (All) N = 414	*P*-Value
Enrollment	0.73 (0.62–0.84)	0.84 (0.72–0.96)	0.77 (0.68–0.85)	0.264
Male n = 26/244				
Female n = 10/134				
4 h	0.71 (0.59–0.82)	0.75 (0.56–0.94)	0.73 (0.63–0.83)	0.750
Male n = 24/187				
Female n = 7/107				
8 h	0.64 (0.49–0.79)	0.74 (0.51–0.97)	0.67 (0.55–0.79)	0.434
Male n = 19/69				
Female n = 9/31				
12 h	0.69 (0.54–0.83)	0.79 (0.58–0.99)	0.71 (0.59–0.83)	0.405
Male n = 19/50				
Female n = 9/25				
16 h	0.67 (0.52–0.81)	0.74 (0.51–0.97)	0.70 (0.58–0.82)	0.622
Male n = 18/44				
Female n = 7/23				
20 h	0.61 (0.46–0.76)	0.63 (0.35–0.91)	0.61 (0.47–0.74)	0.893
Male n = 18/42				
Female n = 8/15				
24 h	0.56 (0.41–0.72)	0.90 (0.77–1.00)	0.64 (0.52–0.77)	0.006
Male n = 16/40				
Female n = 6/16				
36 h	0.57 (0.37–0.77)	0.75 (0.53–0.98)	0.61 (0.46–0.76)	0.280
Male n = 10/27				
Female n = 7/11				
48 h	0.70 (0.50–0.91)	0.65 (0.31–0.99)	0.67 (0.49–0.85)	0.796
Male n = 11/25				
Female n = 5/8				
60 h	0.70 (0.46–0.93)	0.43 (0.9–0.77)	0.60 (0.39–0.80)	0.211
Male n = 9/19				
Female n = 4/7				
72 h	0.71 (0.52–0.91)	0.33 (0–0.78)	0.63 (0.43–0.84)	0.105
Male n = 8/19				
Female n = 3/5				
84 h	0.72 (0.49–0.95)	0.50 (0.05–0.95)	0.65 (0.43–0.87)	0.544
Male n = 8/12				
Female n = 5/1				
96 h	0.80 (0.60–0.99)	n/a	0.63 (0.39–0.87)	–
Male n = 8/13				
Female n = 3/0				
108 h	0.88 (0.63–1.00)	0	0.61 (0.27–0.96)	< 0.001
Male n = 5/4				
Female n = 2/1				
120 h	0.75 (0.43–1.00)	n/a	0.59 (0.26–0.91)	–
Male n = 6/5				
Female n = 2/0				
132 h	0.42 (0.03–0.81)	n/a	0.31 (0–0.63)	–
Male n = 6/2				
Female n = 2/0				
144 h	0.55 (0.18–0.92)	n/a	0.44 (0.11–0.78)	–
Male n = 7/3				
Female n = 2/0				
156 h	0.80 (0.45–1.00)	0	0.57 (0.21–0.94)	< 0.001
Male n = 5/3				
Female n = 2/1				
168 h*	0.96 (0.84–1.00)	n/a	0.75 (0.39–1.00)	–
Male n = 6/4				
Female n = 2/0				

Shown is the performance of UCH-L1 in males versus females.

*Includes results from 180 h.

## Discussion

This prospective study compared the temporal profile and diagnostic accuracy between male and female trauma patients at 20 distinct time-points over seven days of glial and neuronal biomarkers GFAP and UCH-L1. This study is among the first and largest studies to compare the temporal profile of these two biomarkers between sexes in a trauma population in the acute and subacute phase of injury. Since these biomarkers are now commercially available for clinical use it is imperative to assess their performance for any differences that may exist between male and female patients. There were no significant differences in GFAP concentrations between male and female trauma patients but there were significant differences in UCH-L1 levels in the early timepoints post-injury, mostly at enrollment, 4-, 8-, 12-,16-, 20-, and 24-h of injury. These findings were consistent in the longitudinal analysis controlling for age, sex, injury severity score, GCS score, and time. Moreover, this difference held true in both patients with MTBI and trauma without MTBI.

This study represents a mild injury population with 99% of participants having a GCS score of 13–15; 92% having a GCS score of 15; and with a median injury severity score of 4. Furthermore, only 8.7% of the patients had a positive CT. This reflects the population that would benefit most from a blood test to determine the presence of MTBI among trauma patients as well helping to identify those at highest risk of intracranial injury on CT^10^. Although there were 7 patients with GCS scores of 9–12, only 3 had CT lesions. Some patients with 9–12 are often difficult to assess clinically during the first hours after injury if patients are intoxicated, medicated, and in distress.

The pattern of biomarker release in male and female patients was similar, with GFAP detectible within 1-h of injury and reaching a peak at 20-h post-injury. In both sexes concentrations decreased steadily over 72 h and were still detectable at 180-h post-injury. Similarly, the pattern of elevation for UCH-L1 was similar between male and female patients with a peak at 8 h and steady decline over 48 h. However, male patients had significantly higher concentrations of UCH-L1 than female patients at several timepoints post-injury, particularly within 24 h of injury. This pattern of higher elevations of UCH-L1 in male compared to female patients was also seen among males without MTBI, and in males with no evidence of traumatic intracranial lesions on CT. Given that UCH-L1 was significantly higher in males with no head trauma, consideration should be given to following points. Firstly, male patients had a slightly higher injury median injury severity score than female patients 5 versus 4 (although this is not clinically significant). However, after adjusting for potential confounders and examining the results longitudinally, the sex differences in UCH-L1 were still evident. Secondly, UCH-L1 may be very sensitive to very mild trauma. Thirdly, UCH-L1 may be released from other tissues following trauma. It is documented that UCH-L1 is found in testicular tissue^17^ and is elevated cryptorchidism in human males^18^. Therefore, it may be inherently higher in male patients. There are a few studies in the sports literature that have found that male athletes have higher baseline serum UCH-L1 concentrations than female athletes^19,20^. These findings could have implications for adjusting cut-off points for diagnostic tests currently developed or in development.

In terms of diagnostic accuracy, GFAP had higher AUROCs in female patients within 36 h post-injury in both detecting MTBI and detecting CT lesions. Correspondingly, UCH-L1 had higher AUROCs in female patients within 12 h of injury. However, they did not reach statistical significance, indicating that GFAP and UCH-L1 are similarly diagnostic in male and female trauma patients. Therefore, for both male and female patients, GFAP is potentially useful for clinical decision making for detecting a MTBI and also for detecting traumatic intracranial lesions on CT up to 7 days post-injury, whereas UCH-L1’s ability seems to be limited more to the earliest time-points post-injury. This is consistent with previous work in this area^5^.

The authors recognize that there are limitations to this study. This study addressed severity of injury in the acute care setting and did not describe long-term outcome in these patients. The main outcomes used in this study reflect current standards of practice and accepted definitions of acute brain injury severity. All patients presented to a single Level I trauma center in order to assess their performance in a multiple trauma setting. Since this study was at a single trauma center, this may limit the generalizability to other centers, particularly community hospitals. The demographics of our population is, however, very comparable to other trauma centers across the country.

The number of samples available for analysis decreased over the course of the study. This reflects the challenge of obtaining samples over time in patients with less severe injuries because they are not hospitalized as long. However, there were a large number of patients without TBI and patients with mild TBI who were captured in our longitudinal sample because they were admitted for other injuries.

Although the female and male patients had similar in injury patterns and injury severity scores, there may be other injury characteristics not considered in this analysis that could affect the differences in UCH-L1 between the sexes. Additional research is required to replicate these findings in the full spectrum from mild to severe TBI.

## Conclusion

Although the patterns of release of GFAP and UCH-L1 over a week post-injury in trauma patients were similar between the sexes, there were significantly higher concentrations of UCH-L1 in male patients compared to female patients. These higher concentrations of UCH-L1 were most evident within the first 24-h of injury and detected in both MTBI patients and trauma patients without MTBI. Sex differences were not found with GFAP. Despite this, the overall diagnostic accuracies of GFAP and UCH-L1 over time for detecting MTBI and detecting CT lesions was not significantly different between male and female trauma patients. These findings need to be explored further in a larger cohort of patients and in different TBI severities.

## Supplementary Information

Supplementary Information.

## Data Availability

The datasets used and/or analyzed during the current study are not available for sharing at this time but will be available from the corresponding author on reasonable request in the future.
