# Supplementary material for: Sex differences in time course and diagnostic accuracy of GFAP and UCH-L1 in trauma patients with mild traumatic brain injury
Source: Sci Rep. 2023 Jul 22;13:11833. doi: 10.1038/s41598-023-38804-4 (PMC10363108; doi:10.1038/s41598-023-38804-4)
Supplement: Supplementary file 1 — Supplementary Information. [file 41598_2023_38804_MOESM1_ESM.docx]

**eTable 1a.** Mean Concentrations of GFAP in Male versus Female Trauma Patients

|  | **GFAP**  **pg/mL (95%CI)**  **Male Patients**  **N=362** | **GFAP**  **pg/mL (95%CI)**  **Female Patients**  **N=222** | **P-Value** |
| --- | --- | --- | --- |
| **Enrollment** | 180 (116-244) | 142 (76-207) | 0.966 |
| **4 hours** | 247 (165-330) | 176 (98-254) | 0.724 |
| **8 hours** | 673 (377-969) | 632 (360-904) | 0.268 |
| **12 hours** | 903 (485-1321) | 774 (398-1150) | 0.517 |
| **16 hours** | 1004 (555-1454) | 793 (302-1283) | 0.972 |
| **20 hours** | 949 (541-1357) | 1021 (413-1628) | 0.813 |
| **24 hours** | 892 (475-1308) | 765 (281-1249) | 0.777 |
| **36 hours** | 734 (241-1228) | 690 (253-1127) | 0.432 |
| **48 hours** | 513 (147-880) | 405 (100-710) | 0.764 |
| **60 hours** | 326 (44-608) | 229 (39-420) | 0.919 |
| **72 hours** | 157 (36-278) | 119 (8-236) | 0.761 |
| **84 hours** | 138 (31-246) | 181 (50-312) | 0.140 |
| **96 hours** | 118 (25-211) | 134 (8-270) | 0.331 |
| **108 hours** | 117 (8-257) | 64 (8-167) | 0.999 |
| **120 hours** | 83 (8-163) | 80 (8-385) | 0.513 |
| **132 hours** | 71 (10-131) | 66 (8-517) | 0.711 |
| **144 hours** | 86 (13-160) | 71 (8-522) | 0.758 |
| **156 hours** | 70 (8-153) | 44 (8-153) | 0.999 |
| **168 hours** | 30 (8-77) | 81 (8-506) | 0.286 |

*No female patients with samples at 180 hours post-injury

**eTable 1b.** Mean Concentrations of UCH-L1 in Male versus Female Trauma Patients

|  | **UCH-L1**  **pg/mL (95%CI)**  **Male Patients**  **N=362** | **UCH-L1**  **pg/mL (95%CI)**  **Female Patients**  **N=222** | **P-Value** |
| --- | --- | --- | --- |
| **Enrollment** | 872 (726-1018) | 538 (400-675) | **<0.001** |
| **4 hours** | 734 (596-880) | 390 (307-473) | **<0.001** |
| **8 hours** | 822 (632-1011) | 403 (264-543) | **0.002** |
| **12 hours** | 490 (359-622) | 319 (206-431) | 0.126 |
| **16 hours** | 399 (295-503) | 198 (127-270) | **0.016** |
| **20 hours** | 364 (284-445) | 186 (120-252) | **0.015** |
| **24 hours** | 374 (272-475) | 166 (101-232) | **0.015** |
| **36 hours** | 242 (180-305) | 168 (85-251) | **0.040** |
| **48 hours** | 184 (144-225) | 124 (65-183) | **0.030** |
| **60 hours** | 156 (116-195) | 102 (87-117) | **0.049** |
| **72 hours** | 188 (118-258) | 119 (45-196) | 0.115 |
| **84 hours** | 167 (103-232) | 105 (45-174) | 0.348 |
| **96 hours** | 226 (118-334) | 59 (45-103) | **0.008** |
| **108 hours** | 233 (111-355) | 59 (45-102) | **0.004** |
| **120 hours** | 189 (93-286) | 45 (45-45) | 0.103 |
| **132 hours** | 164 (78-249) | 45 (45-45) | 0.089 |
| **144 hours** | 255 (81-430) | 73 (45-45) | 0.121 |
| **156 hours** | 255 (46-464) | 63 (45-142) | 0.085 |
| **168 hours** | 129 (58-45) | 100 (100-100) | 0.643 |

*No female patients with samples at 180 hours post-injury

**eTable 2.** Adjusted Odds Ratios from logistic regression analysis assessing factors associated with sex upon emergency department arrival

| **Characteristic** | **Odds Ratio (95%CI)**  **All Trauma Patients** | **p-value** |
| --- | --- | --- |
| UCH-L1 | 1.55 (1.15-2.09) | **0.004** |
| GFAP | 0.74 (0.50-1.10) | 0.133 |
| Age | 1.00 (0.99 1.01) | 0.604 |
| Injury Severity Score | 1.05 (1.01-1.10) | **0.029** |
| GCS Score on Arrival | 1.13 (0.76-1.70) | 0.542 |
|  |  |  |
| **Characteristic** | **Odds Ratio (95%CI)**  **MTBI Patients** | **p-value** |
| UCH-L1 | 1.39 (1.03-1.87) | **0.031** |
| GFAP | 0.80 (0.54-1.18) | 0.268 |
| Age | 1.00 (0.98 1.01) | 0.837 |
| Injury Severity Score | 1.01 (0.96-1.06) | 0.725 |
| GCS Score on Arrival | 1.19 (0.79-1.78) | 0.416 |
|  |  |  |
| **Characteristic** | **Odds Ratio (95%CI)**  **Trauma Control Patients (no MTBI)** | **p-value** |
| UCH-L1 | 2.71 (1.26-5.84) | **0.011** |
| GFAP | 0.002 (0-7.1) | 0.136 |
| Age | 1.00 (0.98 1.01) | 0.762 |
| Injury Severity Score | 1.13 (1.04-1.23) | **0.006** |
| GCS Score on Arrival* | n/a | n/a |
|  |  |  |

**eTable 3.** Results of Generalized Estimating Equations for repeated measures of UCH-L1 over 7 days with results adjusted for age, sex, injury severity score, and GCS Score among: 1) all trauma patients, 2) MTBI patients, 3) trauma control patients with no MTBI

| **Characteristic** | **Odds Ratio (95%CI)**  **All Trauma Patients** | **p-value** |
| --- | --- | --- |
| Age | 1.01 (0.998-1.004) | 0.429 |
| Sex | 1.22 (1.11-1.34) | **<0.001** |
| Injury Severity Score | 1.03 (1.02-1.04) | **<0.001** |
| GCS Score | 0.96 (0.89-1.04) | 0.358 |
|  |  |  |
| **Characteristic** | **Odds Ratio (95%CI)**  **MTBI Patients** | **p-value** |
| Age | 1.003 (0.998-1.007) | 0.219 |
| Sex | 1.27 (1.11-1.46) | **<0.001** |
| Injury Severity Score | 1.02 (1.01-1.04) | **0.001** |
| GCS Score | 0.86 (0.92-1.07) | 0.861 |
|  |  |  |
| **Characteristic** | **Odds Ratio (95%CI)**  **Trauma Control Patients (no MTBI)** | **p-value** |
| Age | 0.999 (0.996-1.002) | 0.674 |
| Sex | 1.14 (1.02-1.28) | **0.020** |
| Injury Severity Score | 1.02 (1.01-1.04) | **<0.001** |
| GCS Score* | n/a | n/a |
|  |  |  |

*All trauma control patients had a GCS Score of 15

**eTable 4.** Results of Generalized Estimating Equations for UCH-L1 over time with interaction between sex and timepoints adjusted for age, injury severity score, and GCS Score in all trauma patients

| **Characteristic** | **Odds Ratio (95%CI)**  **All Trauma Patients** | **p-value** |
| --- | --- | --- |
| Enrollment | 2.44 (1.92-3.10) | **<0.001** |
| 4 hours | 2.05 (1.64-2.55) | **<0.001** |
| 8 hours | 1.68 (1.35-2.09) | **<0.001** |
| 12 hours | 1.34 (1.09-1.64) | **0.005** |
| 16 hours | 1.24 (1.02-1.51) | **0.028** |
| 20 hours | 1.24 (1.02-1.50) | **0.030** |
| 24 hours | 1.23 (1.02-1.49) | **0.033** |
| 36 hours | 1.12 (0.92-1.34) | 0.286 |
| 48 hours | 1.08 (0.89-1.30) | 0.444 |
| 60 hours | 1.05 (0.88-1.27) | 0.574 |
| 72 hours | 1.06 (0.88-1.28) | 0.557 |
| 84 hours | 1.01 (0.83-1.22) | 0.931 |
| 96 hours | 1.05 (0.87-1.27) | 0.608 |
| 108 hours | 1.13 (0.89-1.44) | 0.310 |
| 120 hours | 1.08 (0.96-1.23) | 0.208 |
| 132 hours | 0.99 (0.86-1.14) | 0.895 |
| 144 hours | 1.16 (0.94-1.43) | 0.160 |
| 156 hours | 1.09 (0.97-1.23) | 0.166 |
| 168 hours | n/a | n/a |

*Results represent Sex*Time Interaction in the GEE model*

**eTable 5.** Results of Generalized Estimating Equations for UCH-L1 over time with interaction between sex and timepoints adjusted for age, injury severity score, and GCS Score in MTBI patients

| **Characteristic** | **Odds Ratio (95%CI)**  **MTBI Patients** | **p-value** |
| --- | --- | --- |
| Enrollment | 3,18 (2.31-4.38) | **<0.001** |
| 4 hours | 2.48 (1.88-3.28) | **<0.001** |
| 8 hours | 2.05 (1.57-2.67) | **<0.001** |
| 12 hours | 1.54 (1.21-1.97) | **<0.001** |
| 16 hours | 1.41 (1.11-1.79) | **0.005** |
| 20 hours | 1.36 (1.08-1.72) | **0.010** |
| 24 hours | 1.34 (1.05-1.69) | **0.016** |
| 36 hours | 1.19 (0.94-1.51) | 0.143 |
| 48 hours | 1.15 (0.90-1.46) | 0.276 |
| 60 hours | 1.13 (0.89-1.43) | 0.308 |
| 72 hours | 1.12 (0.88-1.43) | 0.354 |
| 84 hours | 1.05 (0.82-1.35) | 0.681 |
| 96 hours | 1.10 (0.86-1.42) | 0.453 |
| 108 hours | 1.21 (0.90-1.63) | 0.212 |
| 120 hours | 1.07 (0.91-1.25) | 0.441 |
| 132 hours | 1.03 (0.83-1.27) | 0.822 |
| 144 hours | 1.24 (0.87-1.77) | 0.227 |
| 156 hours | 1.16 (0.91-1.48) | 0.231 |
| 168 hours | n/a | n/a |

*Results represent Sex*Time Interaction in the GEE model*

**eTable 6.** Results of Generalized Estimating Equations for UCH-L1 over time with interaction between sex and timepoints adjusted for age, injury severity score, and GCS Score in trauma control patients with no MTBI

| **Characteristic** | **Odds Ratio (95%CI)**  **Trauma Control Patients (No MTBI)** | **p-value** |
| --- | --- | --- |
| Enrollment | 1.73 (1.56-1.91) | **<0.001** |
| 4 hours | 1.60 (1.46-1.76) | **<0.001** |
| 8 hours | 1.29 (1.15-1.45) | **<0.001** |
| 12 hours | 1.21 (1.07-1.37) | **0.003** |
| 16 hours | 1.19 (1.05-1.34) | **0.006** |
| 20 hours | 1.25 (1.08-1.45) | **0.004** |
| 24 hours | 1.27 (1.11-1.46) | **<0.001** |
| 36 hours | 1.13 (1.03-1.24) | **0.012** |
| 48 hours | 1.11 (1.01-1.22) | **0.040** |
| 60 hours | 1.06 (0.96-1.17) | 0.243 |
| 72 hours | 1.09 (0.99-1.20) | 0.086 |
| 84 hours | 1.08 (1.00-1.17) | 0.054 |
| 96 hours | n/a | - |
| 108 hours | n/a | - |
| 120 hours | n/a | - |
| 132 hours | n/a | - |
| 144 hours | n/a | - |
| 156 hours | n/a | - |
| 168 hours | n/a | - |

*Results represent Sex*Time Interaction in the GEE model*
